# Parenchyma Abundance in Wood of Evergreen Trees Varies Independently of Nutrients

**DOI:** 10.3389/fpls.2020.00086

**Published:** 2020-02-19

**Authors:** Martyna M. Kotowska, Ian J. Wright, Mark Westoby

**Affiliations:** ^1^ Department of Biological Sciences, Macquarie University, Sydney, NSW, Australia; ^2^ Department of Plant Ecology and Ecosystems Research, University of Göttingen, Göttingen, Germany

**Keywords:** hydraulic traits, plant adaptation strategy, plant functional traits, soil phosphorus, tissue fractions, wood anatomy

## Abstract

The abundance of living cells in wood—mainly as interconnected axial and ray parenchyma networks—varies widely between species. However, the functional significance of this variation and its role in plant ecological strategies is poorly understood, as is the extent to which different parenchyma fractions are favored in relation to soil nutrients and hydraulic functions. We analyzed wood tissue fractions of 16 Australian angiosperm species sampled from two nearby areas with similar climate but very different soil nutrient profiles and investigated structure-function links with soil and tissue nutrient concentrations and other plant traits. We expected the variation in parenchyma fractions to influence nutrient concentrations in wood xylem, and to find species with lower parenchyma fractions and accordingly lower nutrient requirements on lower-nutrient soils. Surprisingly, both axial and ray parenchyma fractions were mostly unrelated to tissue and soil nutrient concentrations, except for nitrogen concentration in stem sapwood. Species from low nutrient soils showed higher fractional P translocation from both leaves and sapwood, but little patterning with respect to tissue nitrogen. While species from high and low nutrient soils clearly clustered along the soil-fertility axis, their tissue composition varied independently from plant functional traits related to construction costs and hydraulic anatomy. Our findings imply that there is considerable variation among species in the nutrient concentrations within different parenchyma tissues. The anatomical composition of wood tissue seems unrelated to plant nutrient requirements. Even though xylem parenchyma is involved in metabolic functions such as nutrient translocation and storage, parenchyma abundance on its own does not directly explain variation in these functions, even in co-occurring species. While parenchyma is highly abundant in wood of angiosperm trees, we are still lacking a convincing ecological interpretation of its variability and role in whole-tree nutrient budgets.

## Introduction

Wood is often considered a dead tissue serving the tree mainly for structural stability to lift the photosynthetically active leaves above neighbors competing for light. However, inside the woody xylem a vast interwoven network of living cells remains metabolically active (Sperry, 2003; Evert, 2006) and connected beyond the cambium to processes occurring in the phloem (Sokołowska, 2013; Spicer, 2014). Parenchyma amount can reach values above 40% of wood volume and is highly variable amongst species (Spicer, 2014; Morris et al., 2016) and considerable variability may even occur within species (Fonti et al., 2015; von Arx et al., 2017). Yet we have limited understanding about the costs and benefits of parenchyma abundance in plants (Morris et al., 2016; Carlquist, 2018).

Various physiological functions have been linked to parenchyma including transport and storage of non-structural carbohydrates (Johnson et al., 2012; Plavcová et al., 2016), hydraulic capacitance and vessel refilling after embolism (e.g. De Boer and Volkov, 2003; Secchi et al., 2017), and accumulation of anti-microbial compounds as defense against pathogens and heartwood formation (Spicer, 2005; Morris et al., 2016). Functionality within parenchyma is likely to be influenced by the orientation and connectivity of parenchyma cells—radially as rays, or axially—and additionally by the arrangement type and proximity to tracheary elements—paratracheal or apotracheal—which are commonly used as anatomical classification criteria (Carlquist, 2001). While these distribution patterns have been confirmed for their taxonomic value (Baas, 1982; Wheeler and Baas, 1998), inferences on their functionality often remain indirect or based on morphological observation (Secchi et al., 2017). Independent of the specific function, from a theoretical perspective, the maintenance of these living, cytoplasm-containing cells must be associated with certain costs in terms of photosynthate and soil-derived nutrients. Thus, the variability in nutrient concentration in wood should be linked either with the quantity of living tissue or with the level of metabolic activity in the living cells or with a combination of those two. Indeed, a link between total parenchyma volume in wood and its nutrient concentrations has been suggested in the past (Merrill and Cowling, 1966; De Vries, 1975) and a general pattern of lower nutrient concentrations in gymnosperm wood having low presence of particularly axial parenchyma as compared to angiosperm wood has been described (Meerts, 2002).

Especially when nutrients are expensive to acquire from the soil, plants are expected to develop mechanisms to economize their nutrient budgets by increasing acquisition and reducing nutrient losses. The question how plants mobilize and use nutrients has occupied researchers for nearly a century (Crafts, 1938; Williams, 1955; Vitousek, 1984; Attiwill and Adams, 1993). Adaptations include mechanisms for recycling and internal translocation, and to reduce allocation of nutrients to plant tissue during growth (Lambers et al., 2008). Nutrient translocation prior to senescence is one strategy that allows for nutrient conservation in plants (Brant and Chen, 2015). Previous research has indicated that internal remobilization can contribute a large proportion of the annual nutrient supply required to support the growth of new shoots (Karlsson, 1994; Millard, 1996) and there might be a trade-off between nutrient storage and growth performance in trees (Gleason et al., 2009). It is known that nutrient translocation can occur in all senescing plant parts (e.g. Cherbuy et al., 2001). Nutrient resorption from senescing leaves is a well-studied field providing valuable insights into the nutrient-use efficiency of plants (Killingbeck, 1996; Aerts and Chapin, 2000; Kobe et al., 2005), but our knowledge of P and N mobilization and recycling from stem wood lags far behind that for leaves (Meerts, 2002; Brant and Chen, 2015).

Much research in plant sciences has been focused on nitrogen (N) as the most prominent nutrient determining plant growth in the relatively young postglacial landscapes of Western Europe or North America (Lambers et al., 2008). However, the geologically old and deeply weathered soils in extensive areas of Australia, Africa, and South America are particularly poor in other essential plant nutrients such as phosphorus (P) (Attiwill and Leeper, 1987). Its low availability due to slow diffusion and various processes that lead to P immobilization in the soil (Tiessen, 2008; Shen et al., 2011) make phosphorus a major limiting factor for plant growth (Lambers et al., 2008; Vitousek et al., 2010).

Based on the above considerations we might expect the proportion of sapwood that is parenchyma to covary with sapwood nutrient concentration and also with the degree to which nutrients are withdrawn when sapwood is converted to heartwood, and for all these properties to show patterning in relation to soil nutrient concentrations—especially soil P, in the Australian context. Consequently, in this study we quantified the tissue composition and nutrient concentrations of stem and twig wood from 16 Australian evergreen species, sampled from two nearby sites that differ markedly in soil nutrient concentration. A selection of relevant leaf and wood traits were also measured (e.g. specific leaf area SLA, wood density, and hydraulic conductivity) in order to better understand plant strategies related to nutrient budgets and growth. We hypothesized that: 1) With parenchyma cells being alive and metabolically active, high-parenchyma species would have higher nutrient concentrations in xylem; 2) Species on low-nutrient soils would show lower parenchyma fractions in xylem and by this means have lower nutrient requirements in wood compared to species on high nutrient soils; 3) High-parenchyma species would show pronounced changes in nutrient concentrations during conversion of sapwood into heartwood; and 4), In a manipulative experiment, the response of wood composition to low nutrient supply would be species-specific and therefore not especially plastic or variable within a given species.

## Materials and Methods

### Study Sites and Species

The study region was located in Strickland State Forest near the eastern coast of Australia (151.32°E, 33.38°S). The vegetation is sclerophyll forest on Hawkesbury sandstone with rainforest elements in the gullies on richer soils, e.g. derived from older shale parent materials (Benson and Howell, 1990). Average annual rainfall from 1954–2017 in the closest climate station (Ourimbah, Dog Trap Road: 151.33°E, 33.36°S) was 1,404 mm with an annual mean maximum temperature of 21.8°C and minimum of 11.3°C. During the investigation from March 2017–June 2018, the annual rainfall was 1,263 mm with three consecutive dry months under 8 mm rain in July, August and September 2017.

The 12 study sites were selected based on close proximity, apparent homogeneity, accessibility and occurrence of at least 10 tree individuals of the selected species within a radius of 20m. Six sites were located on the sandstone plateau, while the other six sites were distributed along the gully (**Table 1**). The distance between the sites ranged between 100 to 500 m. We aimed at establishing pronounced differences in soil nutrient contents keeping other environmental variables as similar as possible. However, slight variations in soil organic carbon contents, soil texture and water holding capacity could not be avoided. We chose 16 native evergreen tree species (**Table 2**), eight that were the most common on the nutrient poor sandstone plateau and eight occurring on nutrient rich sites in gullies. The selected species were taxonomically diverse, representing 14 genera from eight families. Of each species five separately growing mature individuals were used as replicates (**Table 3**). Three random soil samples were taken per site to 10cm depth. The samples were oven-dried at 60°C for 48 h and analyzed for total soil P by Vista Pro ICP-OES (Varian, Palo Alto, California, USA) after pseudo-total soil digestion using 100mg of sample digested with a Milestone Ethos-1 microwave digester with aqua regia. Total soil N and C were analyzed with a LECO Truspec CHN combustion analyzer (LECO, Saint Joseph, Michigan, USA). To obtain measures of nutrients more immediately available to plants, P and other soil micro-nutrients were Mehlich-3 extracted for 5 min (Mehlich, 1984), centrifuged and filtered extracts determined using ICP-OES. Soil nutrient analyses were carried out at the School of Agriculture and Food Sciences, University of Queensland.

**Table 1:** Soil carbon content (C), total nitrogen (N_total_), total phosphorus (P_total_), total potassium (K_total_), total Calcium (Ca_total_) and extractable phosphorus (P_extractable_)—of the investigated sites and the soil used for the greenhouse experiment.

	C	N_total_	P_total_	K_total_	Ca_total_	P_extractable_
	%	mg g^-1^	mg kg^-1^	mg kg^-1^	mg kg^-1^	mg kg^-1^
High 1	4.44	3.23	150.5	571.0	283.1	9.90
High 2	4.06	3.57	140.8	8853.7	1344.6	5.03
High 3	3.65	3.42	192.2	6393.0	377.0	5.15
High 4	4.71	3.68	198.9	4709.7	283.3	6.27
High 5	4.76	3.51	122.5	3057.7	425.1	4.73
High 6	5.37	3.01	123.6	317.0	64.0	5.12
Low 1	2.23	0.93	56.3	568.1	243.7	4.52
Low 2	1.32	0.74	48.7	1924.8	27.9	3.16
Low 3	1.20	0.69	45.1	292.4	88.8	1.67
Low 4	1.74	0.81	51.7	2143.4	59.3	3.48
Low 5	1.10	0.71	15.9	1154.4	76.8	0.65
Low 6	1.24	0.78	44.2	1052.9	27.8	2.57
Greenhouse	10.27	1.85	50.6	1281.9	2269.1	12.36

Three soil samples down to the depth of 10 cm were taken and pooled for each site.

**Table 2:** Average nitrogen (N, mg g^-1^) and phosphorus (P, mg kg^-1^) concentrations of tissue samples from 16 tree species sorted by increasing soil nutrient status including fresh leaves, senescent leaves and branch wood, outer sapwood, inner wood and bark.

Species		Family	Nitrogen (N)						Phosphorus (P)					
			mg g^-1^						mg kg^-1^					
			*fresh leaves*	*senescent leaves*	*twig wood*	*outer sapwood*	*inner wood*	*bark*	*fresh leaves*	*senescent leaves*	*twig wood*	*outer sapwood*	*inner wood*	*bark*
*Eucalyptus piperita*	Euc_pip	Myrtaceae	15.73 ± 0.34	10.20 ± 0.55	5.05 ± 0.10	2.00 ± 0.12	3.33 ± 0.51	7.85 ± 0.20	799.1 ± 73.5	312.0 ± 37.1	223.0 ± 15.4	59.6 ± 7.7	59.7 ± 5.5	251.3 ± 15.8
*Angophora costata*	Ang_cos	Myrtaceae	8.67 ± 0.73	5.06 ± 0.25	2.63 ± 0.32	1.87 ± 0.25	0.94 ± 0.07	1.73 ± 0.18	426.6 ± 49.8	106.4 ± 18.0	200.9 ± 42.0	87.8 ± 6.6	12.7 ± 2.7	8.7 ± 0.3
*Banksia serrata*	Ban_ser	Proteaceae	6.91 ± 0.20	3.19 ± 0.53	2.93 ± 0.41	1.61 ± 0.12	0.68 ± 0.09	2.07 ± 0.27	205.1 ± 10.4	62.7 ± 26.5	97.3 ± 11.8	25.6 ± 3.4	8.7 ± 0.3	111.8 ± 9.9
*Eucalyptus haemastoma*	Euc_hae	Myrtaceae	10.01 ± 0.70	5.76 ± 0.77	2.18 ± 0.30	1.38 ± 0.11	0.96 ± 0.19	2.84 ± 0.22	370.6 ± 45.9	125.5 ± 26.2	102.2 ± 20.1	27.3 ± 7.5	8.3 ± 0.08	112.5 ± 19.9
*Banksia ericifolia*	Ban_eri	Proteaceae	8.00 ± 0.33	3.52 ± 0.15	2.01 ± 0.42	1.01 ± 0.12	0.78 ± 0.12	1.70 ± 0.24	159.0 ± 17.6	38.4 ± 2.8	100.5 ± 35.8	8.2 ± 0.4	9.0 ± 0.8	55.4 ± 19.5
*Leptospermum poligalifolium*	Lep_pol	Myrtaceae	12.65 ± 0.24	10.42 ± 0.67	1.68 ± 0.17	0.69 ± 0.08	1.15 ± 0.15	2.08 ± 0.29	551.2 ± 58.3	170.4 ± 85.3	95.8 ± 11.9	24.0 ± 10.2	7.5 ± 0.9	69.0 ± 9.9
*Syncarpia glomulifera*	Syn_glo	Myrtaceae	8.90 ± 0.41	4.83 ± 0.17	3.40 ± 0.14	1.72 ± 0.09	1.30 ± 0.10	3.59 ± 0.15	396.7 ± 109.4	101.2 ± 17.9	158.9 ± 13.2	59.6 ± 7.4	12.2 ± 2.4	63.4 ± 7.6
*Persoonia linearis*	Per_lin	Proteaceae	9.51 ± 0.48	5.38 ± 0.45	1.15 ± 0.08	0.69 ± 0.08	0.87 ± 0.06	1.69 ± 0.33	516.9 ± 42.8	215.9 ± 28.6	107.3 ± 7.8	40.9 ± 22.2	16.2 ± 3.9	77.2 ± 19.7
*Callicoma serratifolia*	Cal_ser	Cunoniaceae	10.74 ± 0.29	7.59 ± 0.44	2.29 ± 0.26	0.97 ± 0.18	0.65 ± 0.05	3.08 ± 0.20	420.6 ± 30.0	202.9 ± 30.8	123.3 ± 9.4	37.6 ± 7.4	15.7 ± 4.0	156.6 ± 15.0
*Ceratopetalum apetalum*	Cer_ape	Cunoniaceae	10.53 ± 0.35	5.55 ± 0.40	1.74 ± 0.12	0.77 ± 0.06	0.74 ± 0.11	2.59 ± 0.04	400.4 ± 34.0	138.6 ± 14.1	105.6 ± 11.6	29.6 ± 3.8	22.2 ± 4.7	128.5 ± 6.3
*Pittosporum undulatum*	Pit_und	Pittosporaceae	12.79 ± 0.44	7.43 ± 0.62	2.04 ± 0.22	1.28 ± 0.09	1.19 ± 0.07	3.88 ± 0.34	1041.8 ± 260.0	312.7 ± 38.8	345.7 ± 162.5	82.4 ± 9.1	94.6 ± 10.1	236.7 ± 13.6
*Syzygium oleosum*	Syz_ole	Myrtaceae	15.57 ± 0.39	11.64 ± 0.57	3.19 ± 0.59	2.83 ± 0.46	1.44 ± 0.05	7.15 ± 0.64	556.1 ± 17.2	422.6 ± 28.9	189.9 ± 20.8	140.7 ± 16.5	83.0 ± 5.5	323.0 ± 29.7
*Synoum glandulosum*	Syn_gla	Meliaceae	13.64 ± 0.50	10.09 ± 0.42	2.47 ± 0.17	2.19 ± 0.25	1.11 ± 0.15	6.37 ± 0.19	511.4 ± 57.2	295.5 ± 44.5	152.9 ± 29.7	50.6 ± 5.1	48.1 ± 15.6	196.4 ± 8.8
*Trochocarpa laurina*	Tro_lau	Ericaceae	11.88 ± 0.48	9.40 ± 0.36	2.90 ± 0.18	1.84 ± 0.11	1.30 ± 0.15	7.13 ± 0.72	364.9 ± 26.8	257.4 ± 29.9	260.8 ± 49.6	106.8 ± 50.3	77.5 ± 8.6	194.5 ± 30.0
*Cryptocarya macrocarpa*	Cry_mac	Lauraceae	8.47 ± 0.33	3.89 ± 0.38	1.47 ± 0.18	1.76 ± 0.39	0.78 ± 0.06	1.73 ± 0.19	243.6 ± 8.7	54.8 ± 17.9	78.9 ± 8.8	26.0 ± 8.2	8.2 ± 0.1	81.4 ± 5.5
*Sloanea australis*	Slo_aus	Elaeocarpaceae	12.86 ± 0.15	9.60 ± 0.30	3.85 ± 0.31	1.22 ± 0.12	1.54 ± 0.19	3.13 ± 0.17	489.0 ± 47.1	394.2 ± 38.6	417.8 ± 101.6	132.0 ± 9.7	130.4 ± 11.8	183.8 ± 10.9

The values are mean ± SE (n = 5). Leaf and twig samples were collected twice during the season in April and October 2017.

**Table 3:** Measured traits of 16 tree species sorted by increasing soil nutrient status – tree structure: diameter at breast height (dbh) and height; hydraulic traits: d_max_ (maximal vessel diameter), dh_mean_ (hydraulically weighted vessel mean), K_S_ (theoretical hydraulic conductivity), A_lumen_ (lumen area); construction cost traits: WD (wood density), SLA (specific leaf area), HV (Huber value), bark thickness, and growth traits: LL (leaf lifespan), BAI (relative basal area increment).

Species		Organ	dbh *cm*	height *m*	*hydraulic traits*				*construction traits*				*growth traits*	
					d_max_	dh_mean_	K_S_	A_lumen_	WD	SLA	HV	bark thickness	LL	rel. BAI
					*µm*	*µm*	*kg m^-1^MPa^-1^s^-1^*	*%*	*g cm^-3^*	*cm^2^ g^-1^*	*cm^2^ cm^-2^*	*mm*	*years*	*% yr^-1^*
*Eucalyptus piperita*	Euc_pip	stem	28.07 ± 4.90	13.80 ± 1.72	128.74 ± 13.75	156.16 ± 10.89	74.28 ± 22.43	10.23 ± 1.47	0.623 ± 0.036			1.17 ± 0.16		5.81 ± 1.68
		twig			71.92 ± 5.01	56.4 ± 4.24	11.05 ± 1.56	12.66 ± 0.32		52.41 ± 7.17	1.49 ± 0.39		3.21	
*Angophora costata*	Ang_cos	stem	38.31 ± 7.22	14.16 ± 1.10	165.94 ± 16.2	160.94 ± 13.72	74.51 ± 25.11	10.17 ± 1.48	0.765 ± 0.028			1.47 ± 0.23		0.58 ± 0.15
		twig			50.13 ± 1.75	41.84 ± 1.80	3.18 ± 0.46	7.16 ± 0.96		66.64 ± 6.51	1.72 ± 0.30		2.33	
*Banksia serrata*	Ban_ser	stem	14.13 ± 1.33	6.42 ± 0.34	90.43 ± 3.99	73.65 ± 3.73	14.24 ± 2.21	11.03 ± 2.27	0.598 ± 0.023			2.47 ± 0.50		0.68 ± 0.42
		twig			48.76 ± 2.57	30.32 ± 1.10	2.51 ± 0.34	10.47 ± 1.13		49.52 ± 0.93	2.94 ± 0.24		2.11	
*Eucalyptus haemastoma*	Euc_hae	stem	17.55 ± 1.96	7.82 ± 0.65	160.21 ± 10.16	195.73 ± 15.31	209.74 ± 32.97	19.97 ± 1.11	0.638 ± 0.014			0.92 ± 0.09		0.64 ± 0.37
		twig			58.38 ± 4.47	48.82 ± 1.55	5.55 ± 0.74	8.52 ± 0.80		31.62 ± 0.88	2.69 ± 0.23		2.60	
*Banksia ericifolia*	Ban_eri	stem	10.47 ± 0.70	5.92 ± 0.55	100.85 ± 3.89	72.57 ± 2.31	13.01 ± 1.62	9.84 ± 0.93	0.474 ± 0.015			0.52 ± 0.04		7.10 ± 1.69
		twig			40.97 ± 2.63	27.39 ± 1.12	1.97 ± 0.32	9.44 ± 0.96		77.29 ± 1.98	2.20 ± 0.33		2.85	
*Leptospermum poligalifolium*	Lep_pol	stem	9.22 ± 1.45	6.88 ± 0.20	64.19 ± 15.58	80.62 ± 3.32	20.20 ± 2.58	11.16 ± 0.73	0.700 ± 0.011			0.57 ± 0.12		3.58 ± 2.12
		twig			49.71 ± 2.71	38.62 ± 1.80	5.37 ± 0.84	13.07 ± 1.24		101.23 ± 3.44	2.07 ± 0.44		1.62	
*Syncarpia glomulifera*	Syn_gla	stem	11.47 ± 1.32	7.47 ± 0.89	133.41 ± 16.65	84.62 ± 3.76	15.66 ± 1.02	8.15 ± 0.50	0.550 ± 0.022			0.56 ± 0.10		2.07 ± 0.38
		twig			46.94 ± 2.14	36.24 ± 1.72	3.89 ± 0.54	11.22 ± 0.83		140.59 ± 10.10	1.07 ± 0.20		3.10	
*Persoonia linearis*	Per_lin	stem	7.26 ± 0.84	5.77 ± 0.53	76.71 ± 13.79	53.98 ± 4.85	7.02 ± 1.13	9.14 ± 0.32	0.565 ± 0.009			1.28 ± 0.25		4.49 ± 2.22
		twig			37.38 ± 1.20	29.75 ± 1.02	2.66 ± 0.35	10.58 ± 0.67		90.22 ± 8.39	1.56 ± 0.10		2.75	
*Callicoma serratifolia*	Cal_ser	stem	12.92 ± 0.80	9.60 ± 0.43	69.26 ± 5.45	49.20 ± 0.62	14.61 ± 1.29	21.5 ± 1.28	0.450 ± 0.001			0.44 ± 0.03		1.13 ± 0.47
		twig			32.02 ± 2.13	27.59 ± 0.96	3.78 ± 0.57	17.59 ± 1.41		118.83 ± 5.15	1.04 ± 0.17		5.56	
*Ceratopetalum apetalum*	Cer_ape	stem	15.03 ± 1.88	14.52 ± 2.23	66.28 ± 4.04	50.70 ± 3.15	10.63 ± 1.34	14.98 ± 0.81	0.530 ± 0.023			0.35 ± 0.01		1.42 ± 0.41
		twig			31.48 ± 2.18	26.66 ± 1.17	2.27 ± 0.26	11.93 ± 1.15		112.73 ± 5.23	1.57 ± 0.13		2.24	
*Pittosporum undulatum*	Pit_und	stem	10.64 ± 0.39	12.06 ± 0.32	73.99 ± 2.28	54.98 ± 2.35	8.85 ± 1.47	10.22 ± 1.06	0.650 ± 0.040			0.27 ± 0.02		0.62 ± 0.23
		twig			41.24 ± 1.67	34.24 ± 1.03	3.27 ± 0.28	10.06 ± 0.36		125.84 ± 3.81	1.25 ± 0.08		2.88	
*Syzygium oleosum*	Syz_ole	stem	11.93 ± 1.16	10.96 ± 0.91	121.39 ± 16.60	74.07 ± 3.09	15.97 ± 2.74	10.48 ± 1.10	0.654 ± 0.026			0.58 ± 0.06		1.11 ± 0.60
		twig			46.62 ± 2.95	37.44 ± 2.07	3.84 ± 0.39	10.65 ± 0.32		110.06 ± 6.92	1.69 ± 0.33		5.58	
*Synoum glandulosum*	Syn_glo	stem	24.70 ± 2.02	14.50 ± 1.33	79.14 ± 7.54	121.38 ± 4.22	59.21 ± 9.27	14.46 ± 1.70	0.700 ± 0.055			2.62 ± 0.20		0.69 ± 0.12
		twig			48.56 ± 1.59	37.59 ± 2.57	4.58 ± 0.96	11.65 ± 1.22		61.98 ± 3.89	1.25 ± 0.23		2.65	
*Trochocarpa laurina*	Tro_lau	stem	10.87 ± 2.38	6.32 ± 1.04	90.74 ± 6.19	44.19 ± 1.72	5.72 ± 0.42	10.62 ± 0.95	0.650 ± 0.023			0.62 ± 0.09		1.30 ± 1.26
		twig			31.63 ± 2.34	24.99 ± 0.88	2.03 ± 0.19	11.38 ± 0.47		120.16 ± 2.13	1.05 ± 0.12		5.83	
*Cryptocarya macrocarpa*	Cry_mac	stem	12.17 ± 1.17	10.86 ± 0.83	101.96 ± 7.60	62.22 ± 2.73	11.97 ± 1.62	11.47 ± 1.01	0.470 ± 0.019			0.36 ± 0.08		1.39 ± 0.52
		twig			15.62 ± 0.99	29.27 ± 1.68	1.91 ± 0.35	8.07 ± 0.96		118.21 ± 5.70	1.14 ± 0.10		4.72	
*Sloanea australis*	Slo_aus	stem	16.27 ± 3.29	15.08 ± 2.87	68.27 ± 6.72	79.67 ± 3.53	17.46 ± 2.67	9.97 ± 0.66	0.536 ± 0.013			0.48 ± 0.07		0.67 ± 0.34
		twig			38.24 ± 3.79	29.44 ± 1.53	1.68 ± 0.28	6.97 ± 0.44		128.69 ± 4.99	1.51 ± 0.19		4.64	

Shown are mean values ± SE (n = 5).

### Plant Material and Sampling

Plant material for nutrient analysis, wood anatomy and trait measurements was collected at the end of austral summer in April 2017 and for twig and leaf samples again in early spring in October 2017. For measuring tissue fractions in xylem, one wood core of the main trunk and one twig sample per tree was taken. The wood cores of length 5–6 cm were extracted using an increment borer with an inner diameter of 5.15 mm (Haglöf, Långsele, Sweden). The samples were transported in marked straws to ensure the determination of inner and outer segments. In the lab the cores were immediately divided into five segments of 1 cm length for sapwood nutrient analysis, sapwood wood anatomy, wood density, inner wood anatomy, and inner wood nutrient analysis (from the outer to the inner wood, respectively). Fresh samples for wood anatomy were stored in 70% ethanol until further processing while nutrient samples were oven dried at 60°C for 48 h. Ethanol storage has proven to be a better storage method based on preliminary work, as cutting previously dried samples led to lower quality sections in some species. For wood density samples the fresh volume was determined using the water-displacement method according to Archimedes' principle with subsequent oven drying at 105°C for 48 h.

For the twig samples one sun-exposed branch per tree optimally in mid-canopy position was chosen and cut using loppers mounted on an elongated pole. From this branch a healthy twig with diameter 5–7 mm was selected and a 3–4 cm segment removed for wood anatomy and for nutrient analysis. All distal leaves were bagged for specific leaf area (SLA, cm^2^ g^-1^) and Huber value (HV; sapwood area over leaf area without bark and pith). From the same branch, 5–30 healthy and intact mature leaves (the youngest fully expanded leaves without any sign of damage) were taken and dust removed using wet tissue. Senescent leaves (usually visually determinable by an altered color and easy to detach) were collected from the same branch or at least the same tree by shaking the tree or touching the leaf by hand. In the lab, bark from nutrient-sample twigs was carefully removed and bagged separately. Small pieces of stem bark were removed using a knife or small chisel. All plant samples were oven-dried at 60°C for 48 h and then ground and analyzed for C+N contents with a CN auto-analyzer (Vario EL III, Hanau, Germany) and for P and other nutrients after HNO_3_ digestion by inductively coupled plasma optical emission spectrometry analysis (Perkin Elmer Optima 5300 DV) at the University of Göttingen, Germany.

### Wood Anatomical Analyses

**Figure 1:**
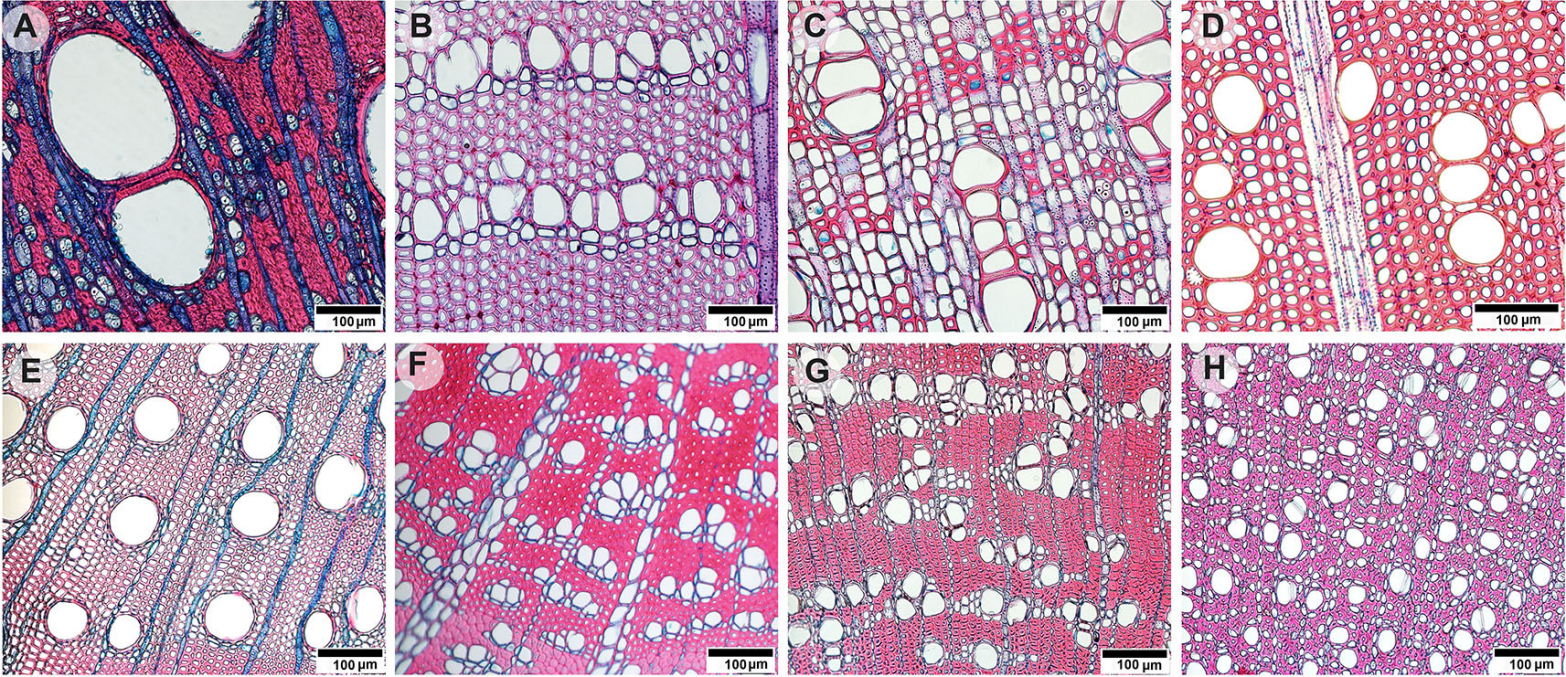
Light microscopy images of transverse stem wood **(A–D)** and twig wood **(E–H)** sections of eight selected evergreen Australian tree species: **(A)** *Angophora costata* with both apotracheal and paratracheal axial parenchyma arrangements, **(B)** *Persoonia linearis* with unilateral paratracheal axial parenchyma and wide ray bands, **(C)** *Synoum glandulosum* with axial parenchyma bands and uniseriate rays, **(D)** *Pittosporum undulatum* with rare axial parenchyma and multiseriate rays. **(E)** *Eucalyptus piperita* with paratracheal axial parenchyma, **(F)** *Banksia ericifolia* with unilateral paratracheal axial parenchyma and wide ray bands, **(G)** *Syzygium oleosum* with both apotracheal and paratracheal axial parenchyma arrangements, and **(H)** *Trochocarpa laurina* with diffuse axial parenchyma. Scale bars indicate 100 µm.

Anatomical trait measures and cell type fractions were obtained from transverse sections of twig and stem wood (**Figure 1**). The 10–25 μm thick sections were cut using a sledge microtome (Reichert, Vienna, Austria) and disposable blades (model A35, Feather Safety Razor Co. Ltd, Japan). Additionally, tangential sections were made for species where the differentiation of axial parenchyma, tracheids, and fibers appeared problematic. Subsequently the sections were stained as follows: staining in 1% safranin (Sigma-Aldrich, Castle Hill, Australia) in 50% ethanol solution for 2 min, washing with distilled water, staining in 1% Astra Blue (Australian Biostain Pty Ltd, Traralgon, Australia) for 30 min, washing with distilled water, and gradually dehydrating over 50% ethanol, 75% ethanol to 99% ethanol, each step taking 2 min. *P. undulatum* and *S. australis* sections were stained in Astra Blue only for 15 min as the coloring was sufficiently saturated after this time. The sections were then mounted in Euparal (BioQuip Products, Rancho Dominguez, USA) and dried at 60°C over 72 h. To determine cell types in detail, high resolution photographs were taken at 200x magnification using a Nikon digital camera (model DXM 1200F, Nikon Corporation, Japan) mounted on a light microscope (Olympus BX 50F, Olympus Co. Ltd., Japan). To analyze cell type fractions and other anatomical traits on larger segments, we took images using a stereo-microscope with an automatic stage equipped with a digital camera (SteREOV20, Carl Zeiss MicroImaging GmbH, Göttingen, Germany) at 125x magnification. Per sample, from 10 up to 40 single images were stitched together to obtain the whole cross-sectional area. Images were processed with Adobe Photoshop CS6 (version 13.0, Adobe Systems Incorporated, USA) and ImageJ (version 1.3e). From each stem section a rectangular shaped area (6–30 mm^2^) was analyzed using as much as possible of the wood core lengths. For twig samples either half or one quarter of each cross section, depending on the quality of the section, was analyzed yielding 1–18 mm^2^ of sapwood area.

To determine cell tissue fractions we used a grid-based counting method as described by Zieminska et al. (2015). Each sample image was overlaid with a regular grid of lines spaced 120–140 μm apart for stem and 40–80 µm apart for twig sections in ImageJ. A minimum 300 intersections for each sample was ensured. Each grid intersection point overlaying the image region was then classified using the “Cell counter” plugin (https://imagej.net/Cell_Counter) depending on which tissue type it was located on, marked and added by tissue type. Anatomical definitions followed “IAWA list of microscopic features for hardwood identification” (IAWA Committee 1989). Cell types were grouped in five categories based on staining, cell wall characteristics and cellular content: 1) conduits: vessel and other conduit lumen, 2) axial parenchyma, 3) ray parenchyma, 4) fiber: fiber lumen together with fiber and conduit wall 5) other: all other cell types including mucilage or oil cells and cell type that could not be determined with certainty. To distinguish between thin-walled fibers and thick-walled axial parenchyma, section fragments were inspected with higher magnification and tangential sections were consulted to assess whether the axial cells have a spindle-shaped morphology typical for fibers or are rectangular and arranged in strands as typical for axial parenchyma. Septate fibers are known to occur in three of our study species, namely *Pittosporum undulatum, Synoum glandulosum,* and *Sloanea australis*. These cells play a role in starch storage and can remain nucleated at maturity in the outer xylem (Rajput and Rao, 1999; Carlquist, 2015; Carlquist, 2018). Here, we included septate fibers under category (4), as their abundance is difficult to quantify accurately from transverse sections and the determination of their metabolic activity requires molecular techniques, radioactive stains or high-resolution microcopy.

Other anatomical traits measured were determined using the particle-analysis function from ImageJ for estimating vessel density (*VD*, n mm^-1^), the idealized vessels diameter (*d*) as *d* = ((32 × (*a* × *b*)^3^)/(*a* ^2^ + *b* ^2^))^¼^, with major (*a*) and minor (*b*) vessel radii, and cumulative vessel lumen area (*A_lumen_*, m^2^). Individual vessel diameters (*d*) were used to calculate the hydraulically weighted mean vessel diameter (*d_h_*) according to Sperry et al. (1994) as *d* = Σ *d* ^4^/*d* ^5^. For these measurements all vessels of a cross section were analyzed, yielding 60 to 3000 measured vessels per species and organ. The theoretical hydraulic conductivity (K_h_) was calculated based on Hagen-Poiseuille's law as *K_h_* = (*π* × Σ *r* ^4^)/8*η*) × *ρ*, with *r* the vessel radius, *η* the viscosity (1.002×10^-3^ Pa s) and *ρ* the density of water (998.2 kg m^-3^), both at 20°C. Theoretical area-specific hydraulic conductivity (*K*_S_, kg m^-1^ MPa^-1^s^-1^) was obtained from *K*_h_ by dividing through the microscopically determined cross-sectional area without bark and pit.

### Plant Trait Measurements and Growth

Leaf area was measured using an Epson Scanner 1680 and ImageJ software (Version 1.50e). For each sample 15 to 100 leaves were scanned depending on the leaf size (more leaves for smaller-leaved species). Scanned leaves were then oven-dried at 60°C for 48 h and weighed for calculation of SLA. Nutrient recycling efficiencies (NRE) for P and N are the proportion of N and P pools translocated before leaf shedding, considered on a mass basis, and are calculated according to Vergutz et al. (2012) as: 1-(conc_senescent_/conc_fresh_). To account and correct for changes in leaf mass during senescence, we calculated SLA for senescent leaves as suggested by van Heerwaarden et al. (2003). However, for these 16 species we did not find a substantial trend of mass reduction as the SLA values of senescent leaves were either very similar to the fresh leaves or slightly higher or lower without following a consistent pattern.

Stem diameter growth dendrometers (UMS, München, Germany) were installed at the beginning of the study in March 2017 on all sampled trees and additionally on three to five other tree individuals to ensure that wood core sampling did not influence stem increment growth. Stem increments were read every 2 months until May 2018 to obtain annual basal area increment (BAI). Additionally, the diameter at 130 cm above the ground (diameter at breast height, DBH) and the height of each tree was measured using a laser vertex (Haglöf Vertex Ultrasonic Hypsometer).

Leaf demography was monitored from May'17–May'18 to obtain leaf birth and shedding. Leaf lifespan was then calculated per species as the mean of these two variables pooled over all branches. For each species at least ten well-developed twigs from 5–9 individual trees were randomly selected in a sun exposed but easy to reach position optimally at the mid-crown of the marked individuals. Occasionally large individuals were bent over and held for the duration of the counting. For each of these twigs a detailed map drawing was created carefully marking and counting green, senescent and dry leaves at each sampling date – May'17, July'17 and May'18 (Milla et al., 2004). Diameter growth at the twig base and at three additional marked locations along the twig was recorded also.

### Greenhouse Experiment

A greenhouse experiment was conducted in parallel to field measurements to investigate the potential for plastic responses of tissue fractions in wood to soil nutrient availability. We selected four of the 16 species that were available as seedlings from commercial nurseries (*Angophora costata, Syncarpia glomulifera, Callicoma serratifolia, Pittosporum undulatum*). Seedlings had all been grown from seed for 5–7 months depending on the species. Seven plants per species and treatment were randomly selected and planted individually into 15 L pots filled with low nutrient native soil (details see **Table 1**). In a low nutrient treatment no additional nutrients were added, while a high nutrient treatment received 3.2g per kg soil of slow-release fertilizer pellets (Osmocote^®^, Scotts Australia, with N:13, P:4.8, K:9.1) intermixed with the soil. This resulted in an addition of 150 µg/g P which is in the range of the total P soil values in our more fertile study sites. Plants were kept in the greenhouse and watered for 2 min two times each day. Plant height and stem diameter growth were monitored each month. After 6 months the largest and smallest were discarded from the seven plants grown for each treatment, and the remaining five were harvested by separating stem and branches, roots and leaves. A sapwood sample of 4–5mm diameter under the bark was taken from each plant. Wood anatomical measurements were performed on this sample as described above.

### Statistical Analysis

All statistical analyses were performed using the statistical software R v. 3.3.2 (R-Core-Team, 2017). For a descriptive data exploration, we calculated Pearson correlations tables and used panel plots to characterize the relationships among traits using the mean trait value per species. Parenchyma fractions were related to soil nutrients, tissue nutrient concentrations and recycling efficiencies using mixed effect linear models (*lmer* package: “*lme4*” and *lm* package: “*stats*”) with species treated as random factor. For the mixed effect models we calculated R^2^ values (*r.squaredGLMM* package: “*MuMIn*”) as: R^2^_marginal_ which describes the proportion of variance explained by the fixed factor alone, and R^2^_conditional_ which includes both the fixed and random factors (Barton, 2018). P-values were obtained by parametric bootstrapping (*PBmodcomp* package:”*pbkrtest*”). Predicted random effects and residuals of the models were checked for normal distribution and homoscedasticity using diagnosis plots and dependent variables were log-transformed when necessary which was only the case for vessel sizes and *K_S_*. For pairwise comparisons of nutrient concentrations and tissue fractions in stem and twig wood within species we used Kruskal-Wallis-tests (*kruskal.test*, package: “*stats*”). To obtain variance partitioning of cell type fractions and functional traits, we tested the effects of species on each parameter with analysis of variance (ANOVA). We calculated the proportion of the variance explained by species and random effects as σ^2^_effect/_σ^2^_total_ x 100.

Principal component analysis (PCA) used *prcomp* from the *“stats”* package to summarize associations among traits and habitat properties using all individual data (n = 80). The variables used for the PCA were tissue fractions, soil N, soil P, *SLA, WD, HV*, bark thickness, *DBH*, *K_S_*, *d_max_*, leaf N+P, stem N+P, N+P recycling. To test the best predictor for parenchyma fractions we applied stepwise backward selection of the most parsimonious model with the “maximum likelihood” method using the *stepAIC* function of the “*MASS*” package (Venables and Ripley, 2002), the model with lowest *AIC* (Akaike information criterion) score being the most parsimonious (Burnham and Anderson, 2002).

## Results

### Parenchyma Fractions and Nutrients

**Figure 2:**
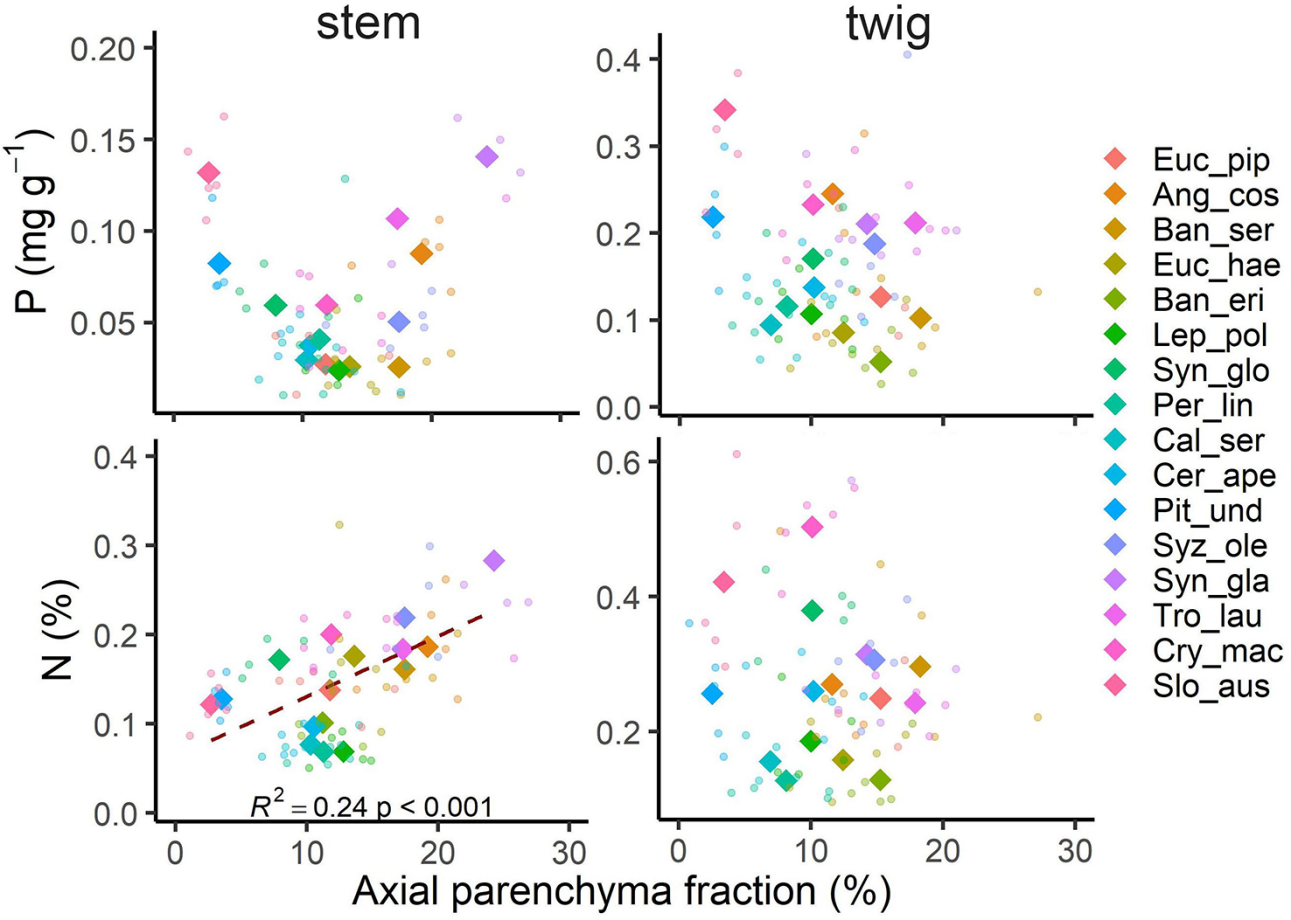
Axial parenchyma fraction in relation to phosphorus (P) and nitrogen (N) concentrations in stem and twig xylem of 16 Australian evergreen species. Shown are species means as rhombus (n = 5) and all data points as circles (n = 80). A significant relationship was only found between N in stem wood and axial parenchyma fraction. Species abbreviations are shown in **Table 2**.

**Figure 3:**
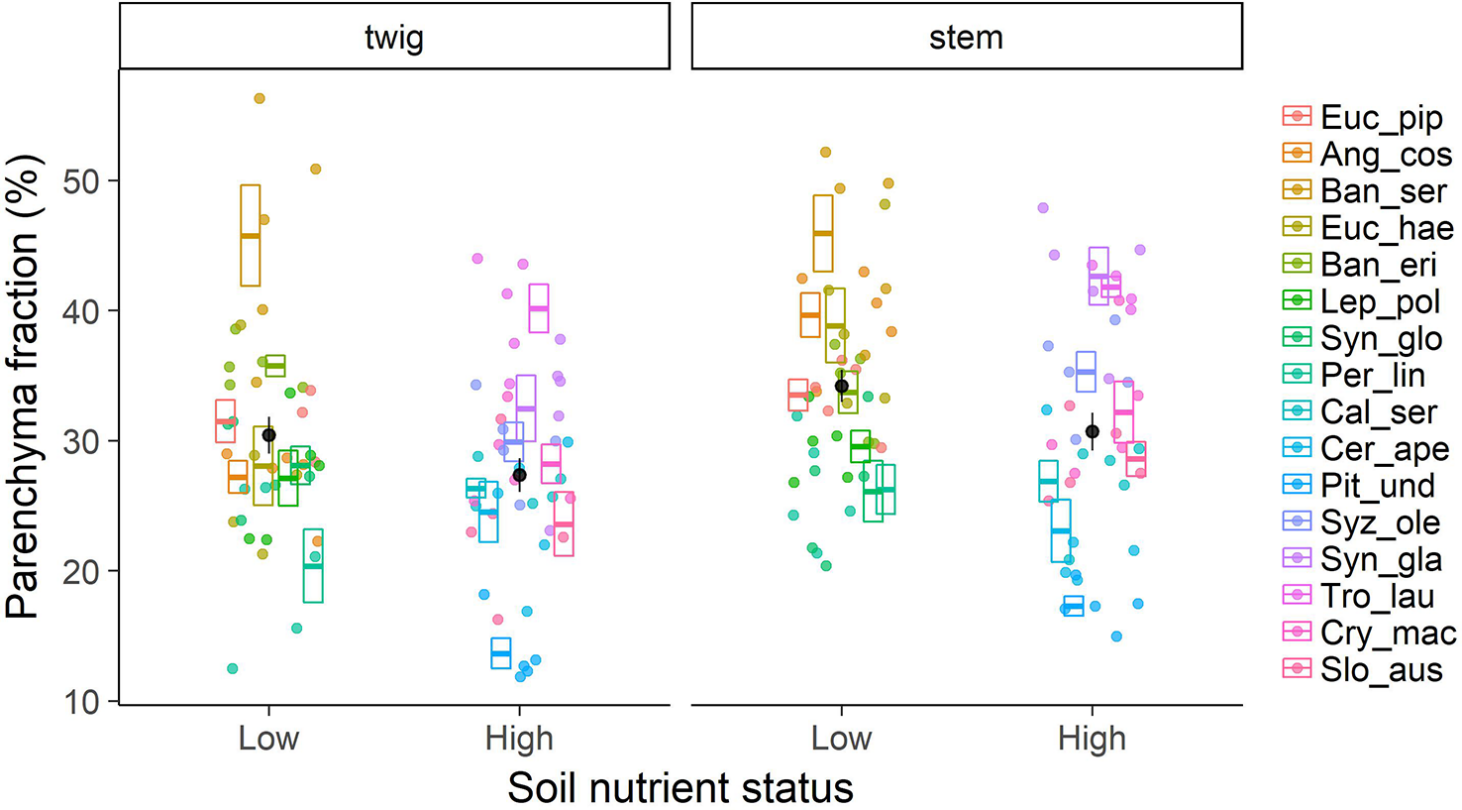
Total parenchyma fraction in xylem wood of 16 Australian evergreen tree species by soil nutrient status (low and high). Boxplots show mean values per species ± SE (n = 5 for each species, total = 80), individual data are depicted as points in the respective colour. Black dots represent mean values ± SE of species groups on low nutrient vs. high nutrient soil.

Contrary to our first hypothesis, nutrient contents in wood were only partly related to the fraction of parenchyma in the tissue (**Figure 2**). For nitrogen (N) there was a positive relationship with total parenchyma fraction in stem wood, but not in twig wood. The relationship was even stronger when we considered axial parenchyma only (p < 0.001; **Table S1**). For phosphorus (P) there was no significant relationship with axial or total parenchyma in either stem or twig wood (p > 0.05; **Table S1**). The expression of nutrient contents on a mass or volume basis did not significantly shift the relationship in either case, nor did excluding the three species—*Pittosporum undulatum, Synoum glandulosum* and *Sloanea australis*—containing septate fibres. Total parenchyma fractions were not significantly lower in species on low nutrient soils, and rather tended to be higher (**Figure 3**). However, there was no significant correlation with soil nutrients either as total N content or available P. This was similar in twig wood and in main stem (p > 0.05; stem: R^2^_m =_ 0.02, R^2^_c_ = 0.74; twig: R^2^_m =_ 0.02, R^2^_c_ = 0.73).

Pronounced differences in parenchyma fractions between species were the most obvious feature of the results (**Figure 3**). The scatter in parenchyma fractions between species was wide, 14.6%–44.9% in stem wood and 13.7%–45.8% in twig wood (**Supplementary Figure 1**), and the variance was largely explained by species identity (69.4% for twigs and 71.3% for stems). Similarly, 60%–80% of variation in all other wood tissue fractions was explained by species identity (**Supplementary Figure 2**).

### Nutrient Recycling Efficiencies

**Figure 4:**
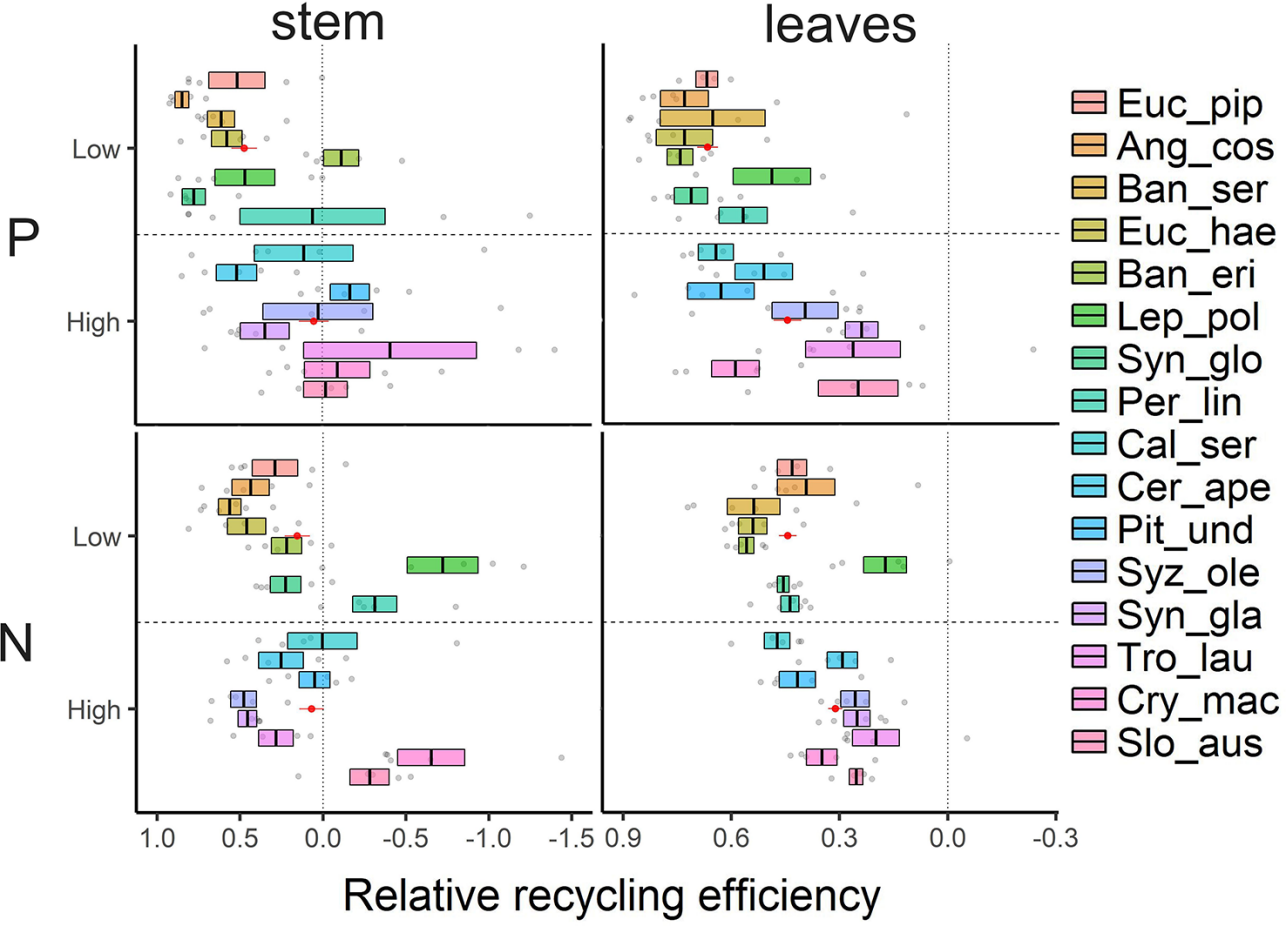
Relative nutrient recycling efficiencies (P, N) calculated as proportion of nutrient pools translocated before tissue senescence of leaves and stem wood. Values of 16 tree species are sorted by increasing soil nutrient status (top to bottom). Shown are mean ± SE (n = 5) and the mean value of species groups on low nutrient vs. high nutrient soil in red as mean ± SE (n = 40).

Similar to tissue nutrient concentrations and in contrast to our third hypothesis, neither N nor P recycling efficiencies from sapwood were correlated with total parenchyma fractions (**Supplementary Figure 3**). Surprisingly, we found negative N or P recycling efficiencies in stem wood of several species, i.e. nutrient concentrations in heartwood were higher than in sapwood. Species involved were *Banksia ericifolia, Leptospermum poligalifolium, Persoonia linearis, Pittosporum undulatum, Trochocarpa laurina, Cryptocarya macrocarpa,* and *Sloanea australis* (**Figure 4**). Overall, species on low nutrient soils recycled P from sapwood more efficiently than species on richer soils (t-test; t = -2.73, p < 0.05). The equivalent effect for N was not significant. Recycling efficiencies were more closely correlated with the nutrient contents in litter or inner wood (proficiency) than with nutrient contents in fresh and functioning leaf or sapwood (**Supplementary Figure 3**). Regarding leaves, P was recycled well in our species, with relative recycling efficiency values up to 77% (**Figure 4**). Species on low nutrient soils tended to recycle P from leaves more efficiently than species on richer soils (t = -3.77, p < 0.05). N recycling rates in leaves were more variable among the investigated tree species, varying from 17%–56% in low nutrient soil and from 20%–47% in higher nutrient soils not showing a significant difference between the soils (t = -0.34, p = 0.74). However, our lower detection limit of P ranged around 0.01 ppm. For five species on low nutrient soils the concentrations in inner wood samples were below this detection limit and we had to assume a value just below the detection limit (detection limit—blind value). Therefore, the recycling efficiencies and proficiencies might potentially be even higher than reported.

### Links to Other Functional Traits

**Figure 5:**
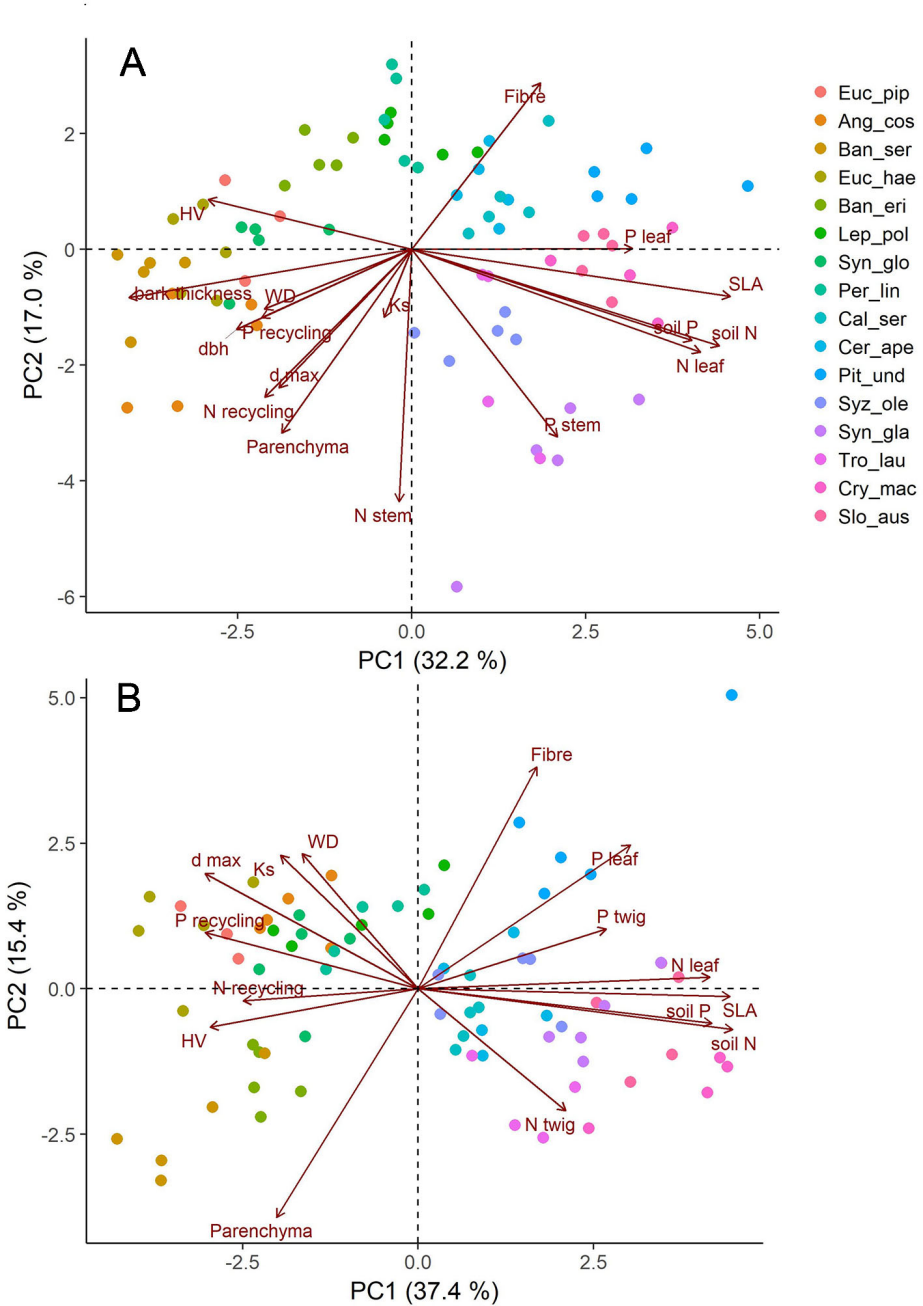
Graphical display of the first two axis of principal component analyses (PCA) with anatomical (fibre and parenchyma fraction), hydraulic (Ks, d_max_), and structural (dbh, WD, SLA) trait data as well as nutrient contents (leaf, wood) and recycling efficiencies; **(A)** traits measured for stems, **(B)** traits measured for twigs of the same individuals. Shown are all data with five replicates of each of the investigated 16 species.

Across the species studied here, none of the other measured traits was correlated with parenchyma fractions in a functional way (**Table 3**, **Supplementary Figure 3**). In a principal component analysis (PCA, **Figure 5**), tissue fractions scaled almost orthogonally to the functional traits WD, SLA, and bark thickness together with leaf and soil nutrient contents. The first two axes of the PCA explained 49.6% of variation in traits of twigs and 49.3% of stems. Accordingly, stepwise regressions with species as a random effect reinforced that most of the measured functional traits did not explain variation in parenchyma well. The best fitting model to explain total parenchyma fractions in stems included just *N_sapwood_*, *height* and *dbh* (R^2^_c_ = 0.68, R^2^_m_ = 0.26). The equivalent model for twigs retained *bark thickness*, *P_leaf_* and *P recycling efficiency* (R^2^_c_ = 0.70, R^2^_m_ = 0.13). Overall, hydraulic traits such as theoretical conductivity (K_S_), maximal vessel diameter (d_max_), hydraulically weighted vessel diameter (d_h_) and lumen area (A_lumen_) also did not correlate very well with parenchyma fractions. There was only a weak link between d_max_ and parenchyma fraction (**Supplementary Figure 4**).

### Plasticity in Tissue Fractions

In a greenhouse experiment we tested how plastic tissue fractions might be in response to varying soil nutrient contents. Growth performance and root-shoot ratio of all four species were considerably affected by nutrient availability. However, according to our expectation, there was no consistent shift in tissue fractions of newly developed xylem. Parenchyma fraction increased in one species, decreased in another, and showed no trend in two (**Supplementary Figure 5**). This lack of consistent plastic response reinforces the interpretation that the tissue fractions in field-grown plants vary largely because of intrinsic differences between species, rather than due to physiological response to soils.

## Discussion

### Linking Parenchyma Abundance to Nutrients

Our results confirmed that parenchyma fractions vary widely across 16 Australian evergreen tree species—up to sevenfold for axial parenchyma and twofold for rays. Similar patterns were observed for twig and stem wood. Contrary to our expectations, the observed variation in parenchyma abundance was not strongly related to nutrient concentrations or nutrient recycling efficiencies in sapwood. However, we found a relationship between N and parenchyma fraction in stem wood, similar to early reports on temperate trees (Merrill and Cowling, 1966), but no association with P. Respiratory and metabolic activity has been shown to vary spatially and temporally across parenchyma cells, particularly for vessel-associated cells (Sauter et al., 1973; Alves et al., 2004; Spicer and Holbrook, 2007). The maintenance of a certain metabolic activity level and thus nutrient concentration throughout the parenchyma network can be expected, due to its anatomically and functionally integration that enables the transports and accumulation of assimilates and nutrients as well as the production of secondary compounds (Morris et al., 2018b).

Vessel-associated parenchyma cells are characterized by a high nucleoplasmic ratio and mitochondrial activity and are expected to influence transport to and from sap-conducting cells (Fromard et al., 1995; Secchi et al., 2017). They may be involved in vessel refilling and surfactant production (Brodersen and McElrone, 2013; Schenk et al., 2017; Secchi et al., 2017). Axial parenchyma can also serve as a storage site for nitrogen-based compounds, either as mobile N reserves or as storage proteins and other live cell constituents (Magel, 2001; Carlquist, 2007). The organic P-pool in plants is typically comprised of phosphate esters in nucleic acids (Ticconi and Abel, 2004; Veneklaas et al., 2012), while excess free inorganic orthophosphate (P_i_) can be stored in cell vacuoles including parenchyma cells (Ticconi and Abel, 2004; Pratt et al., 2009). Ray parenchyma constitutes an interwoven, dynamic system involved in transport processes mainly from the phloem (Kuhn et al., 1997; Chaffey and Barlow, 2001; Ache et al., 2010; Pfautsch et al., 2015). Besides parenchyma, xylem tissue chiefly consists of conduits and fibers constructed from lignified secondary cell walls, themselves composed of polymer cellulose and a variety of hemicelluloses (Sjostrom, 1993; Butterfield, 1980) which can be expected to have a relatively consistent nutrient composition.

In this study the wide variance in xylem nutrient contents could only be weakly explained by parenchyma abundance and other measured variables. The likeliest explanation seems to be that nutrient quantities within parenchyma cells can vary more widely than parenchyma abundance within and across species with pronounced intrinsic differences in storage capacity and metabolic activity ranging down to dormancy. Higher variability in twig wood nutrient concentrations as compared to stem wood can be expected as parenchyma cells in twigs are more directly involved in source-driven nutrient translocation and in storage related to leaf senescence (Aerts and Chapin, 2000; Saur et al., 2000; Sette et al., 2013). Next to parenchyma cells, septate fibers can potentially also show an extended longevity (Carlquist, 2013; Carlquist, 2015; Carlquist, 2018), but in our case excluding the species where septate fibers occur from the analysis did not influence the results.

We expected plants growing on low nutrient soils to have lower parenchyma abundance and wood that is lower in nutrients, since *de novo* nutrient uptake from soil is costly and the maintenance of metabolically active cells requires respiratory investment. Nonetheless, soil nutrient status did not influence the observed variance in fractions of parenchyma cells in woody xylem. Among the 16 co-occurring evergreen species, total parenchyma fractions tended if anything to be higher rather than lower in species on low nutrient soils. As we did not find strong differences in parenchyma fraction across species in relation to soil nutrients under field conditions, plasticity within a species rather than innate differences between species seems an unlikely explanation. Confirming this, we found inconsistent responses of cell type composition to soil nutrient availability across four species in the greenhouse experiment. Unlike hydraulic conductivity and wood density, which can be developmentally influenced by nutrient availability (e.g. Bucci et al., 2006), parenchyma fractions within species at least at a regional range remain unchanged with varying nutrient supply.

### Recycling Efficiency in Leaves and Wood as Related to Nutrients

Even though parenchyma cells are key players in mobilization and translocation of nutrients in wood, their abundance was not related to the net balance between recycling and deposition in this study. A large proportion of parenchyma present in xylem might be expected to increase the amount of soluble compounds susceptible to leaching and translocation (Cornwell et al., 2008). Indeed, decreased nutrient concentrations of N and P (Penninckx et al., 2001) and other trace elements (Riitters et al., 1991) in older wood as compared to outer sapwood have been reported for temperate trees and eucalypts (Laclau et al., 2001; Crous et al., 2019). Reports for nutrient recycling from wood are far less common than from leaves (Meerts, 2002; Brant and Chen, 2015). This is unfortunate, as wood constitutes a larger potential nutrient pool particularly for large trees (Martin et al., 2014; Sardans and Peñuelas, 2015). Similar to leaves, wood nutrients can scale positively with nutrient concentration in the topsoil, and usually decrease with wood depth (Heineman et al., 2016). The same was true for most nutrient concentrations in this study. Nevertheless, the wood recycling efficiencies observed here showed a wide scatter. Heartwood formation in some species is associated with tylosis formation or gum deposition containing polyphenols such as tannins, flavones and quinones which can alter the chemical composition and nutrient content substantially (Meerts, 2002; De Micco et al., 2016). These chemical changes in heartwood primarily aim at increasing the resistance to fungal wood decay (Cornwell et al., 2009), which may play an important role in several of the study species. Thus, heartwood formation can actually increase the construction costs of wood in terms of nutrients instead of being a recycling source.

Besides the down-regulation of nutrient requirements for growth and maintenance, another potential plant adaptation to low soil nutrient availability is to recycle nutrients efficiently within the plant (Vergutz et al., 2012). We found that species on low nutrient soils had on average higher nutrient recycling efficiencies, particularly for translocation from senescing leaves. This trend however seems not to be general as no clear relation between nutrient availability and percentage nutrient translocation was shown in a global study (Aerts, 1996). While our N and P leaf recycled fractions were within the range of reported data from global literature reviews (Aerts, 1996; Killingbeck, 1996; Vergutz et al., 2012), most of our species showed high efficiencies similar to those reported previously for Australian species from P-impoverished ecosystems (Wright and Westoby, 2003; De Campos et al., 2013). Indeed, in our study P was recycled more efficiently than N from both leaves and stems, which was also observed for *Acacia* species on P-poor soil (He et al., 2011) and forest species of Catalonia (Sardans and Peñuelas, 2015). Further the resorption proficiency e.g. the amount of nutrient remaining in senescent tissue is substantially lower in species on poor soils which was observed in this study similarly to previous work (Wright and Westoby, 2003). Increased nutrient retention time *via* longer leaf lifespan has been suggested to be a more efficient way of nutrient conservation than varying resorption efficiency (Escudero et al., 1992). However, we found no relationship between soil nutrient status or recycling efficiency and leaf longevity. Perhaps general patterns of recycling efficiencies on a large scale ought not to be expected, since ecological strategies regulating recycling are likely to be influenced also by factors such as water availability (Lü et al., 2012) and the uptake costs of each specific nutrient.

### Anatomical and Hydraulic Traits

The role of parenchyma in xylem has also been linked to hydraulic functioning (Johnson et al., 2012; Schenk et al., 2017; Olano et al., 2017). As previously shown in a global meta-analysis across >800 species (Morris et al., 2018a), we observed that maximal vessel size was related to axial and total parenchyma fraction. Vessel-associated axial parenchyma cells have been reported to play a prominent role in maintaining long-distance water transport and preventing or even repairing embolism (Braun, 1984; Brodersen and McElrone, 2013; Secchi et al., 2017; Morris et al., 2018b). Parenchyma cells may also play a role in sapwood capacitance (Tyree and Zimmermann, 2002; Meinzer et al., 2003). In general, species from tropical ecosystems tend to show higher parenchyma abundance than temperate species (Baas, 1982; Morris et al., 2016) while also often having greater conduit diameters (Wheeler et al., 2007; McCulloh et al., 2010). In our dataset large trees tended to have higher parenchyma fractions overall, which was also mirrored in stem wood tending to have more parenchyma than twigs. As large trees tend to optimize their water transport capacity through vessel size (Olson and Rosell, 2013; Olson et al., 2014), the relationship between vessel dimensions and parenchyma amount in wood could be at least partly driven by maximal potential tree size (Morris et al., 2018a) and thus indirectly follow hydraulic adjustments.

Nonetheless, hydraulic efficiency can be adjusted independently of changes in parenchyma abundance, as highly conductive wood has been shown to be constructed of different combinations of both ray and axial parenchyma fractions (Zieminska et al., 2013; Zieminska et al., 2015). Similar to Zieminska et al. (2015) we observed a trade-off between parenchyma fraction and fiber fraction across all species, independent of vessel parameters and wood density. Another dimension may exist next to the well-known construction cost and efficiency trade-off in woody xylem which is likely to be related to defense or storage functions in wood. Accordingly, we found that anatomical traits in our study scattered nearly orthogonally to the soil nutrient and leaf economic spectrum axis, which was represented by leaf nutrient concentrations and SLA. Substantial spatial and temporal diversity in the functions of particularly axial parenchyma may be the cause of the difficulties to link any particular variable with its abundance (Carlquist, 2015). Axial and ray parenchyma have been reported to have opposite correlation patterns with wood density and climate variables (Martinez-Cabrera et al., 2009) or being uncorrelated with climate variability and other environmental factors (Zieminska et al., 2015), while ray parenchyma abundance was linked to climate signals in tree rings of a gymnosperm species (Olano et al., 2013). These results underline different functional roles axial and ray parenchyma may have in woody xylem (Zheng and Martínez-Cabrera, 2013) even though both form a three dimensionally-interconnected morphological and most likely functional continuum across the entire symplastic network (Chaffey and Barlow, 2001; Morris et al., 2018b).

Although parenchyma fraction varies from very rare to 20%–40% across species (Wheeler et al., 2007; Morris et al., 2016) and is vital for survival of the trees, we are still far from fully understanding adaptive reasons for this variability. Measuring the type and distribution of structural and non-structural nutrients using integrated molecular and biophysical techniques within cells of different parenchyma types along the morphological continuum might shed light on the question of evolutionary drivers of wood compositional variation and parenchyma development. More detailed examination of physiological functions should be linked with wood anatomical observations (Secchi et al., 2017; Carlquist, 2018). Traits such as decay-avoiding compound deposition should also be investigated, to understand how investment in structural tissues changes plant adaptation strategies and how this confers advantage in environments with low nutrient availability.

## Data Availability Statement

All datasets generated for this study are included in the article/**Supplementary Material**.

## Author Contributions

MK, IW, and MW developed the research question and study design. MK conducted the data acquisitions and analysis in close collaboration with MW. The article was drafted and written by all three coauthors.

## Conflict of Interest

The submitted work was carried out in the absence of any personal, professional, or financial conflict of interest.
