# Supplementary material for: Parenchyma Abundance in Wood of Evergreen Trees Varies Independently of Nutrients
Source: Front Plant Sci. 2020 Feb 19;11:86. doi: 10.3389/fpls.2020.00086 (PMC7045414; doi:10.3389/fpls.2020.00086)
Supplement: Supplementary file 1 [file DataSheet_1.docx]

**Supplementary Material**

**Tables**

Table S1: Parameter estimates of the linear mixed effects models between nutrients in woody xylem and given parenchyma fraction. Given are fixed effect estimate, the upper and lower 95% confidence interval (CI), the standard deviation (Spec.SD) and the residual standard deviation (Res.SD). In case of a statistically significant effect the parameters are depicted in bold which was determined by parametric bootstrapping (P bₒₒₜ(χ²)). Additionally, the marginal and conditional R² (explained variance without and with random species effects) are shown.

|  |  |  | Fixed effect | | Random effect | |  |  |  |
| --- | --- | --- | --- | --- | --- | --- | --- | --- | --- |
| **Model** | | ***n*** | ***Estimate*** | ***95% CI*** | ***Spec.SD*** | ***Res.SD*** | ***R*^2^_m_** | **R^2^_c_** | **P bₒₒₜ(χ²)** |
| *N ~ Axial p.* | |  |  |  |  |  |  |  |  |
|  | *branch* | 78 | -0.0023 | -0.0081 - 0.0035 | 0.101 | 0.181 | 0.01 | 0.61 | 0.46 |
|  | *stem* | **76** | **0.0056** | **0.0029 - 0.0083** | **0.043** | **0.044** | **0.24** | **0.63** | **< 0.001** |
| *P ~ Axial p.* | |  |  |  |  |  |  |  |  |
|  | *branch* | 77 | -0.0021 | -0.007 - 0.0027 | 0.066 | 0.071 | 0.01 | 0.47 | 0.40 |
|  | *stem* | 76 | 0.0015 | -0.0005 - 0.0035 | 0.038 | 0.033 | 0.04 | 0.63 | 0.15 |
| *N ~ Total p.* | |  |  |  |  |  |  |  |  |
|  | *branch* | 78 | 0.0005 | -0.0031 - 0.00427 | 0.102 | 0.081 | 0.001 | 0.61 | 0.75 |
|  | *stem* | **76** | **0.0035** | **0.0014 - 0.0055** | **0.048** | **0.044** | **0.17** | **0.62** | **< 0.01** |
| *P ~ Total p.* | |  |  |  |  |  |  |  |  |
|  | *branch* | 77 | -0.0016 | -0.0046 - 0.0013 | 0.067 | 0.071 | 0.02 | 0.48 | 0.30 |
|  | *stem* | 76 | 0.0003 | -0.0011 - 0.0018 | 0.039 | 0.031 | 0.001 | 0.60 | 0.68 |
| *N ~ Ray p.* | |  |  |  |  |  |  |  |  |
|  | *branch* | 78 | 0.0033 | -0.0021 - 0.0088 | 0.100 | 0.080 | 0.02 | 0.61 | 0.24 |
|  | *stem* | 76 | 0.0005 | -0.0023 - 0.0034 | 0.056 | 0.045 | 0.001 | 0.61 | 0.74 |
| *P ~ Ray p.* | |  |  |  |  |  |  |  |  |
|  | *branch* | 77 | -0.0020 | -0.0065 - 0.0026 | 0.069 | 0.070 | 0.01 | 0.49 | 0.41 |
|  | *stem* | 76 | -0.0008 | -0.0027 - 0.0011 | 0.039 | 0.031 | 0.01 | 0.62 | 0.44 |

**Figures**


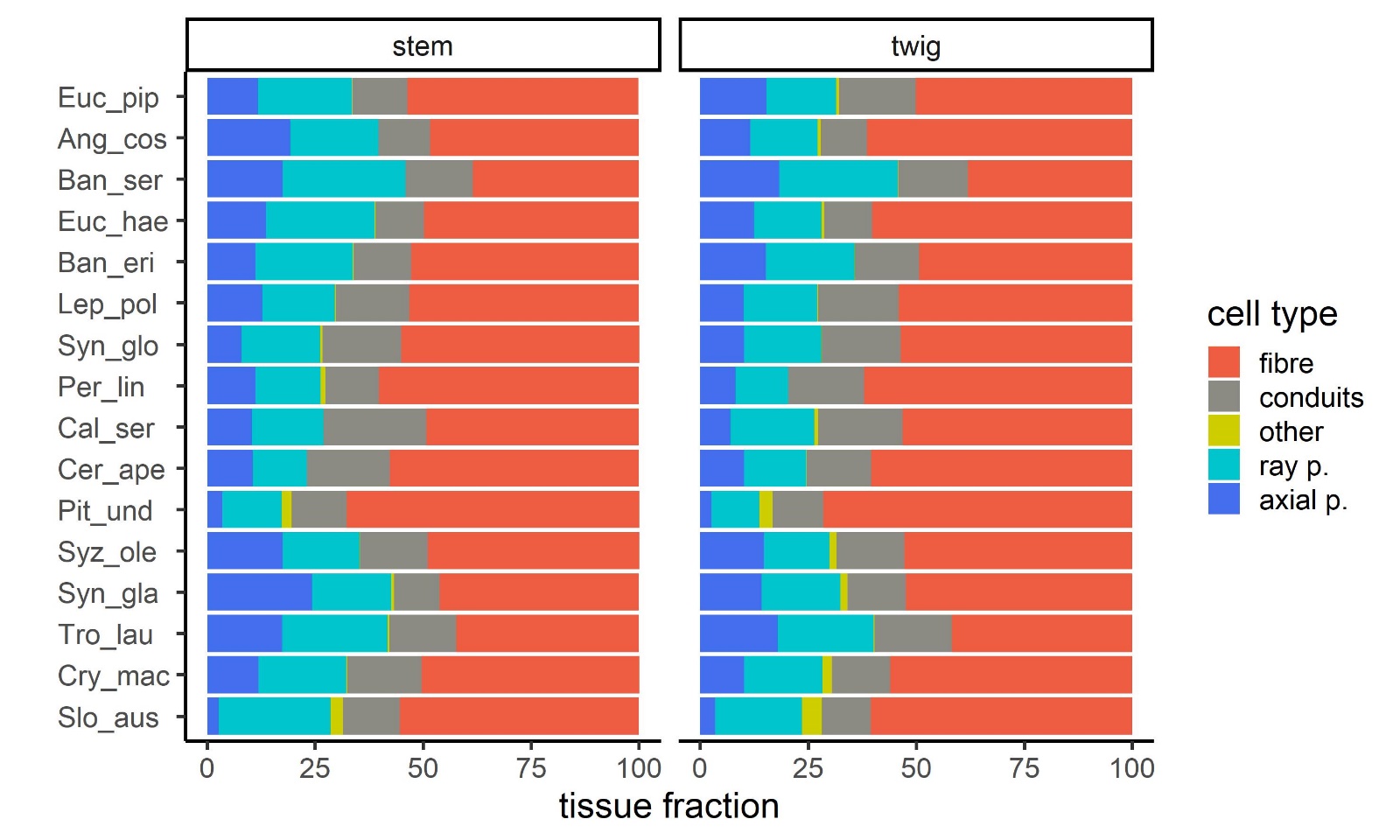


S1: Tissue fractions in stem and twig xylem of 16 Australian evergreen tree species arranged in order of increasing soil nutrient status (from top to bottom). Shown are mean values (n=5).


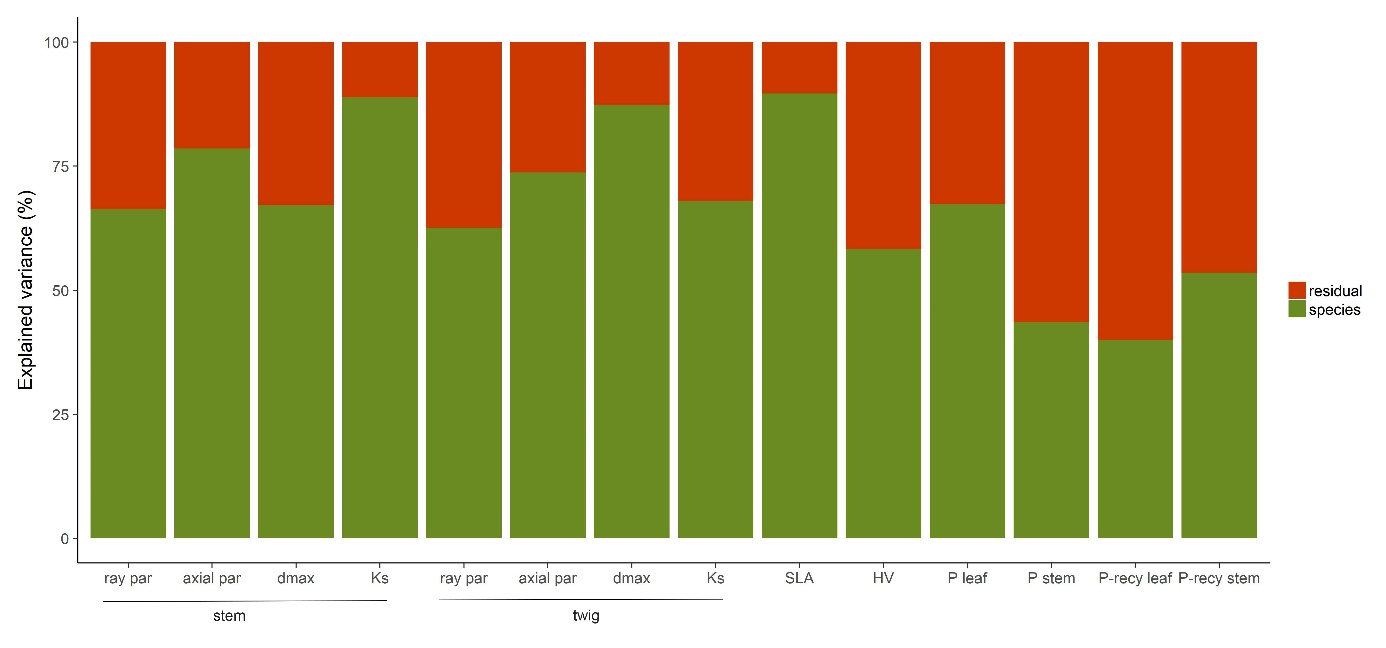


S2: Explained variance by species identity of selected traits – anatomical traits (ray parenchyma fraction = ray par, axial parenchyma fraction = axial par), hydraulic traits (maximal vessel diameter = d_max_, theoretical conductivity = K_S_), construction cost traits (specific leaf area = SLA, Huber value = HV, bark thickness) and nutrients (leaf phosphorus content = P leaf, stem phosphorus content = P stem, P leaf recycling efficiency = P-recy leaf, P stem recycling efficiency = P-recy stem).


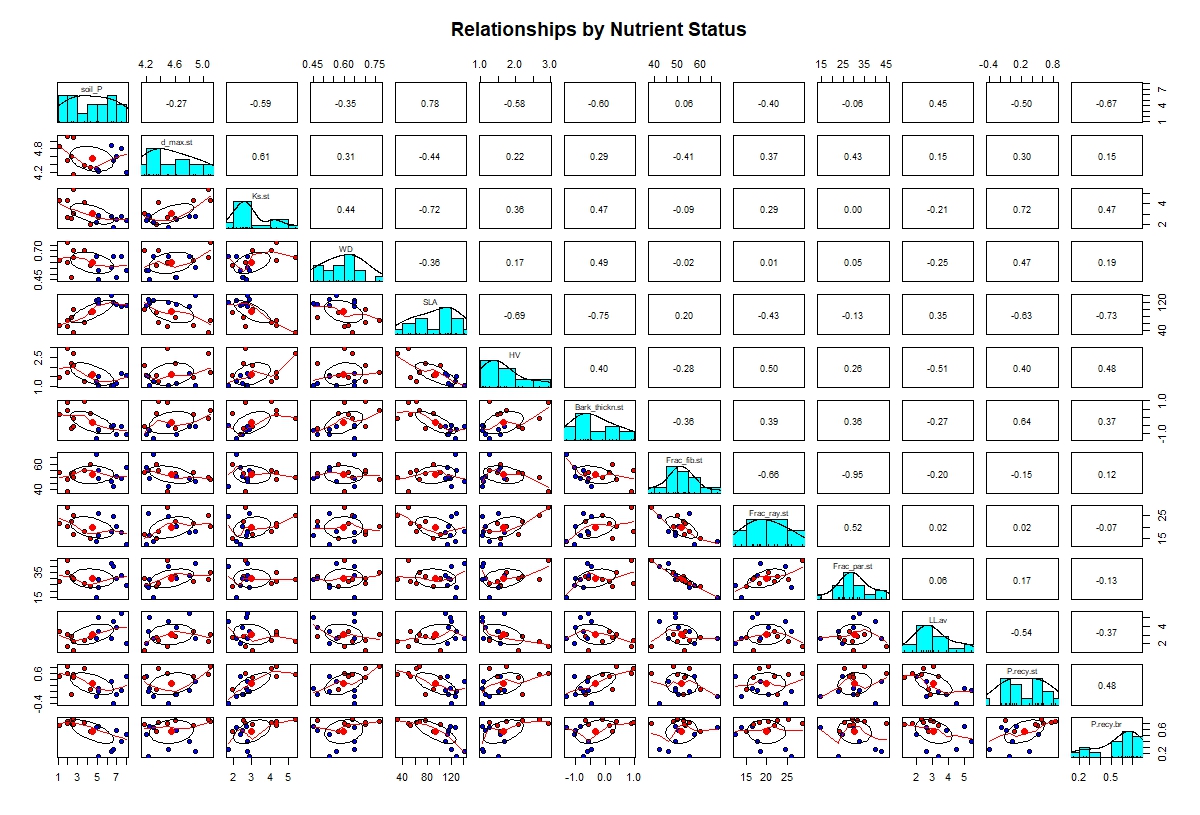


S3: Panel plot of selected traits based on species means (n=5). Red dots indicate species occurring on high nutrient sites and blue dots represent species from low nutrient sites. Soil P content, hydraulic traits (stem maximal vessel diameter = d___max.st, stem theoretical conductivity = K_S_), construction cost traits (specific leaf area = SLA, Huber value = HV, bark thickness, leaf lifespan =LL) anatomical traits (stem fibre fraction = Frac.fib.st, ray parenchyma fraction = Frac.ray.st, total parenchyma fraction = Frac.par.st), and nutrients (P leaf recycling efficiency = P.recy.br, P stem recycling efficiency = P.recy.st).


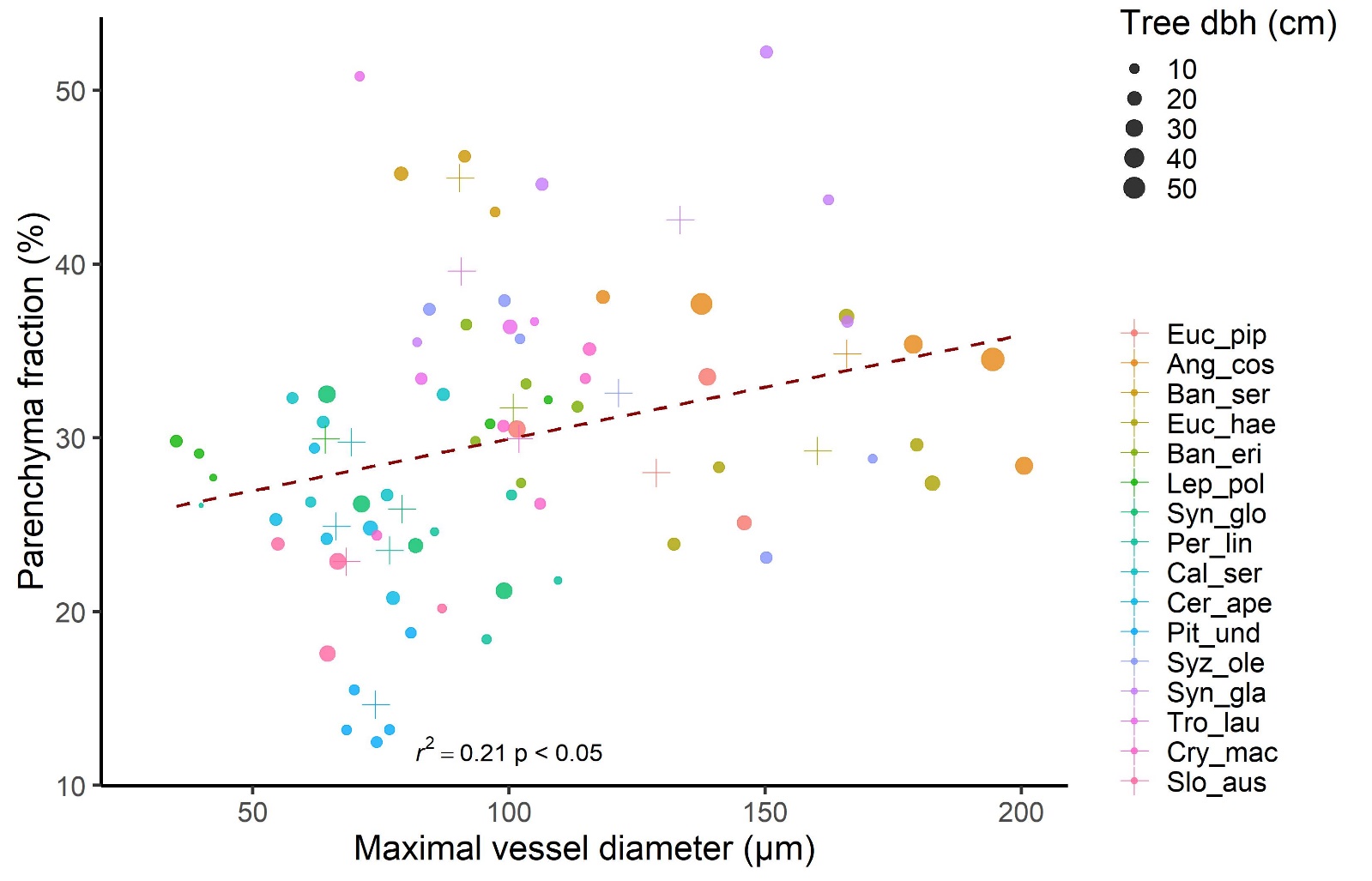


S4: Relationship of parenchyma fraction with maximal vessel size in stem wood of 16 evergreen species. Point size reflects the tree diameter at breast height (dbh) and species mean values are depicted with cross symbols.


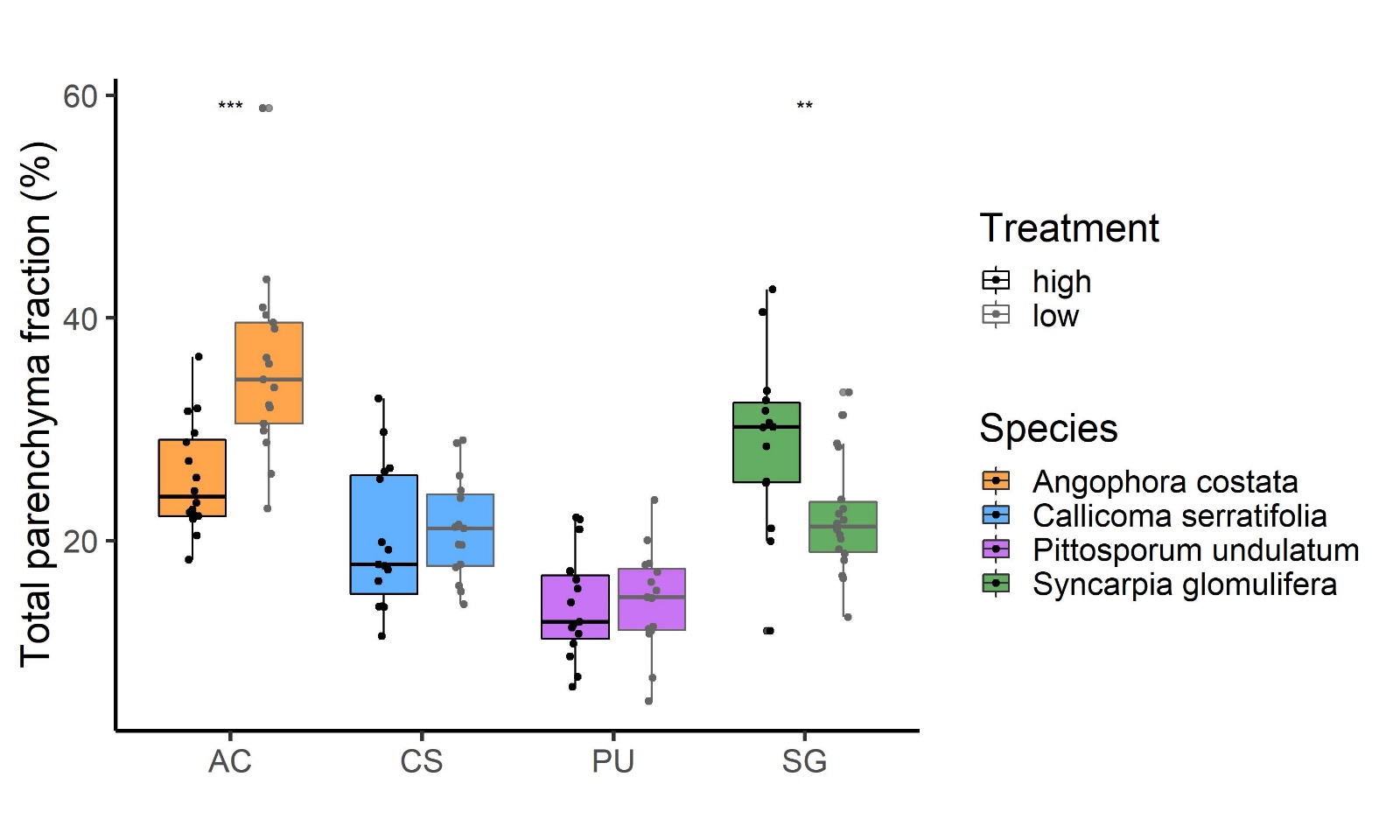


S5: Parenchyma fractions of four tree species grown under high or low soil nutrient supply. Shown are mean values ± SE for each species and treatment (n = 5). We found no difference in total parenchyma amount for the two species which grew well in both treatments - *Callicoma serratifolia* and *Pittosporum undulatum* - and opposing trends of nutrient availability in the two species where the low nutrient-treated plants performed rather poorly - *Angophora costata* and *Syncarpia glomulifera*.
